# Supplementary material for: Histo-molecular differentiation of renal cancer subtypes by mass spectrometry imaging and rapid proteome profiling of formalin-fixed paraffin-embedded tumor tissue sections
Source: Oncotarget. 2020 Nov 3;11(44):3998–4015. doi: 10.18632/oncotarget.27787 (PMC7646834; doi:10.18632/oncotarget.27787)
Supplement: Supplementary file 1 [file oncotarget-11-3998-s001.pdf]

Histo-molecular differentiation of renal cancer subtypes by mass spectrometry imaging and rapid proteome profiling of formalin-fixed paraffin-embedded tumor tissue sections

SUPPLEMENTARY MATERIALS

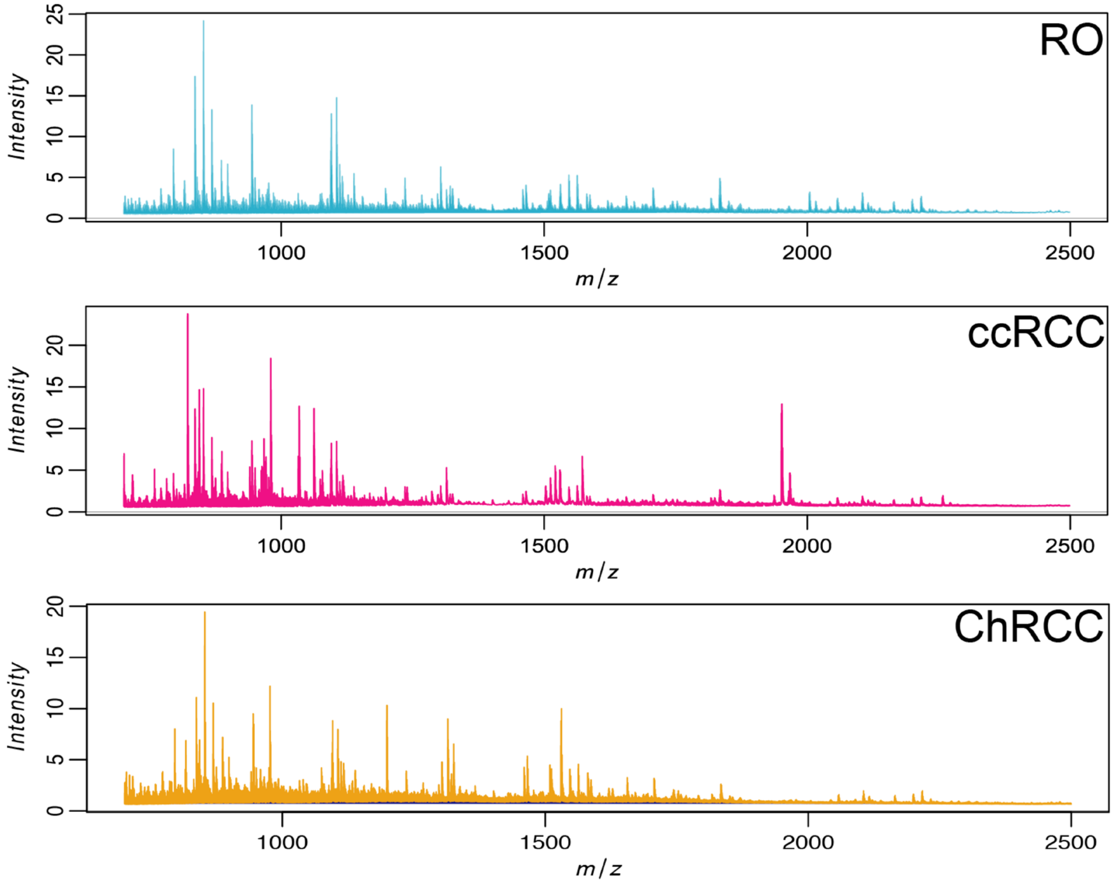

Supplementary Figure 1: Averaged MALDI spectra of respective cancer types. Average MALDI spectra obtained for each of the individual cancer types. Average spectra are based on the extracted pixel. The spectra of the 3 cancer types show differences in their average spectra. However as the pixel amount/patient sample is varying obvious differences in the average spectra can be misleading when looking for distinctive features.

Indicates tumor area

Site of extraction (Note the size of the circle is not correlated to the actual extraction area)

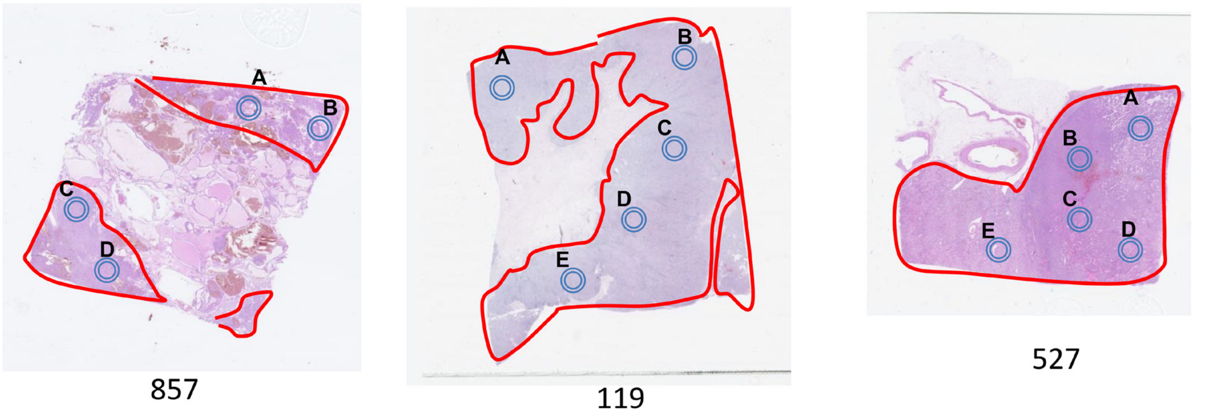

Indicates tumor area

Site of extraction (Note the size of the circle is not correlated to the actual extraction area)

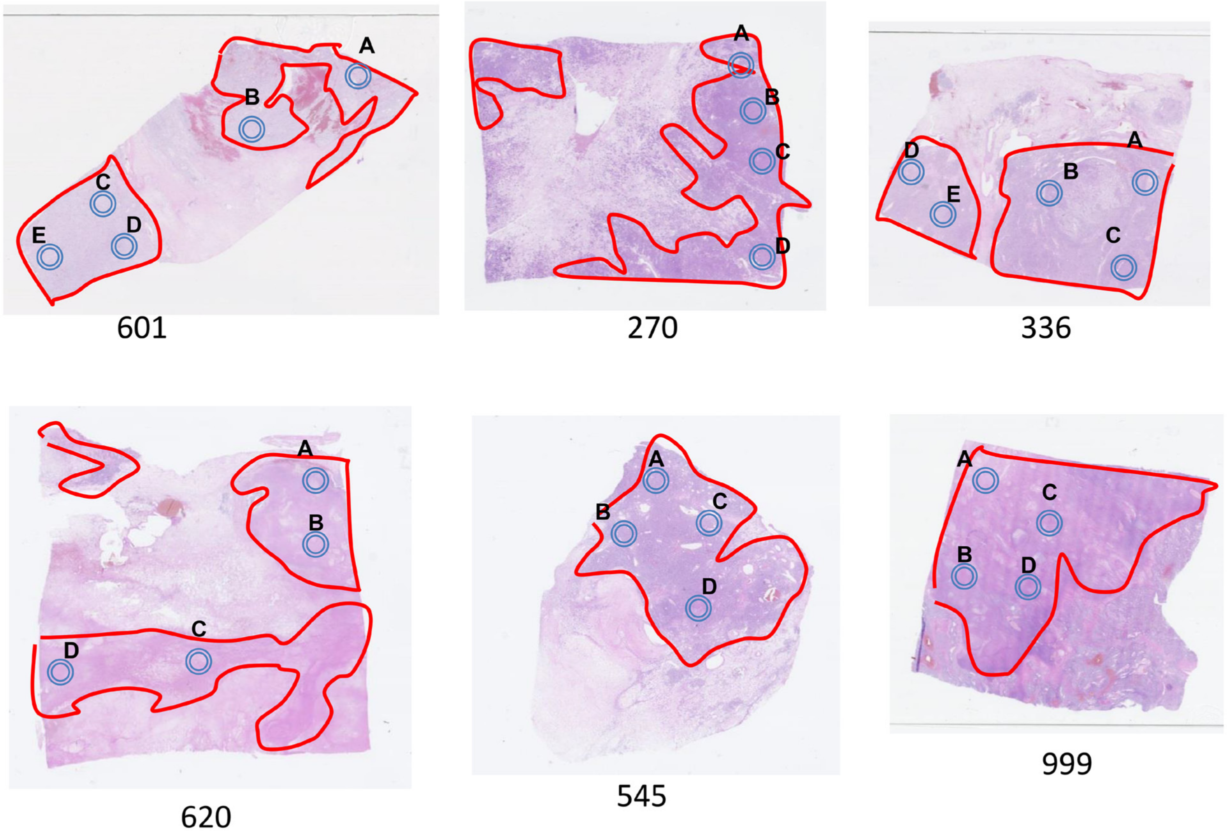

Indicates tumor area

Site of extraction (Note the size of the circle is not correlated to the actual extraction area)

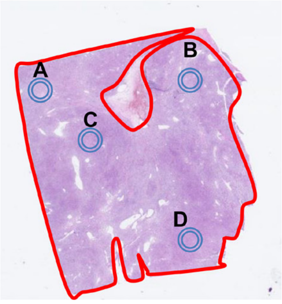

073

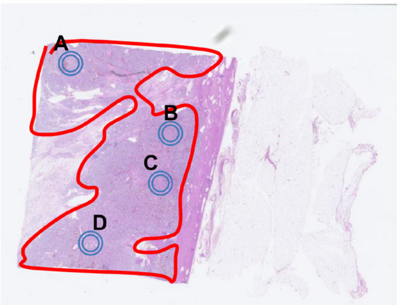

370

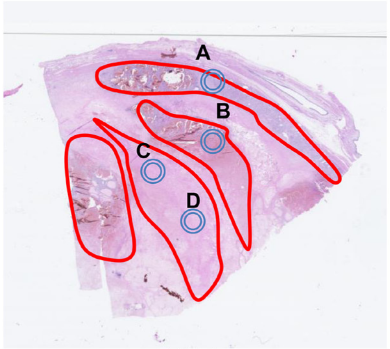

797

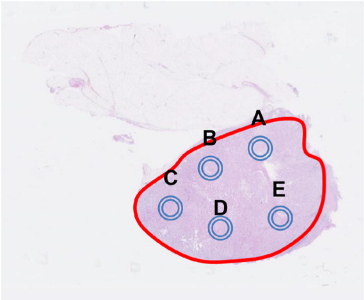

427

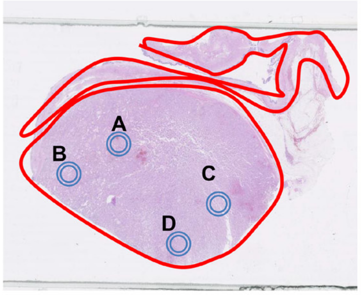

839

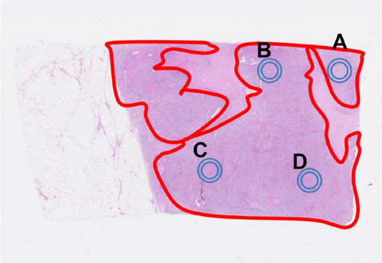

560

Indicates tumor area

Site of extraction (Note the size of the circle is not correlated to the actual extraction area)

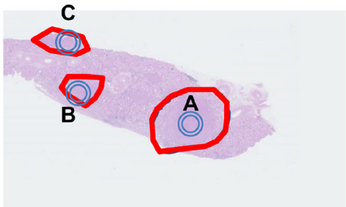

924

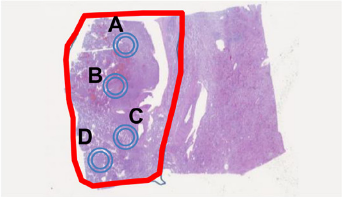

725

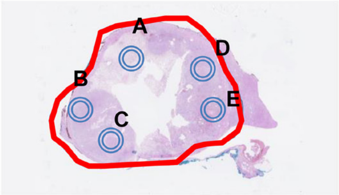

310

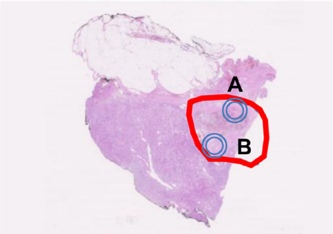

853

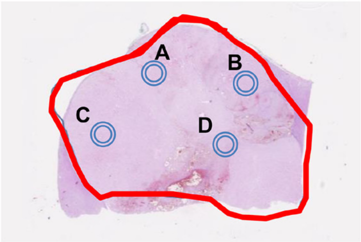

756

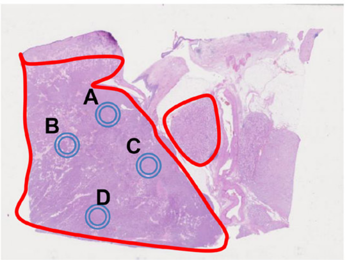

529

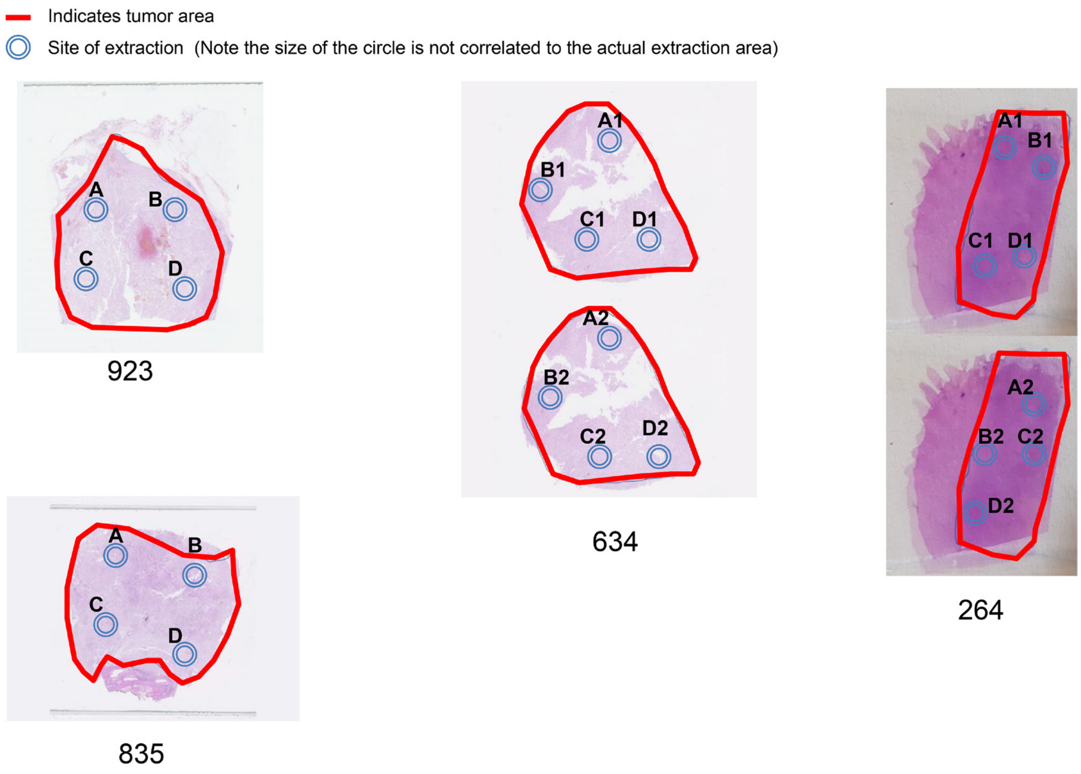

Supplementary Figure 2: HE-stains of FFPE sections used in this study.

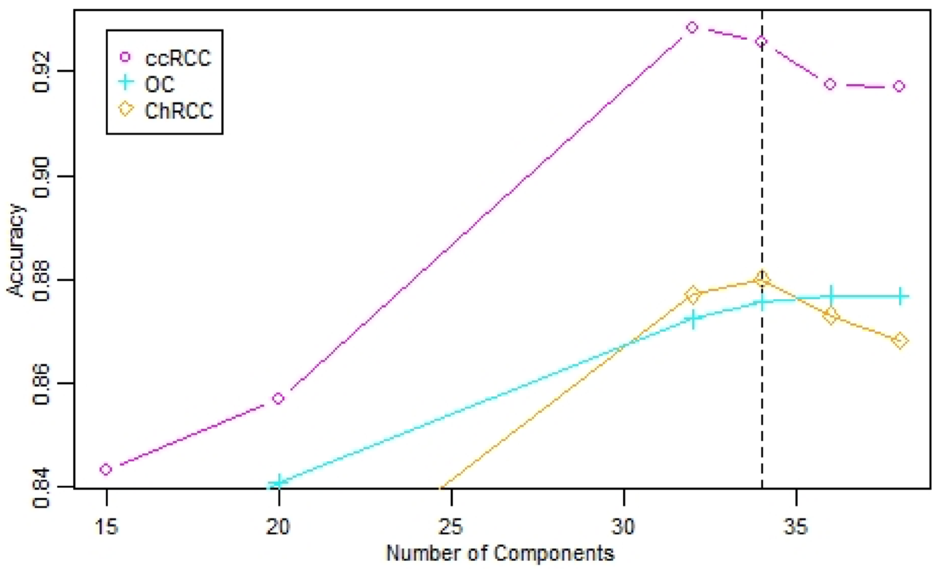

Supplementary Figure 3: Parameter optimization for PLS-DA. Development of prediction accuracy with different PLS-DA-components. ccRCC RO and ChRCC. Chosen optimum n-components = 34 is marked by dotted line.

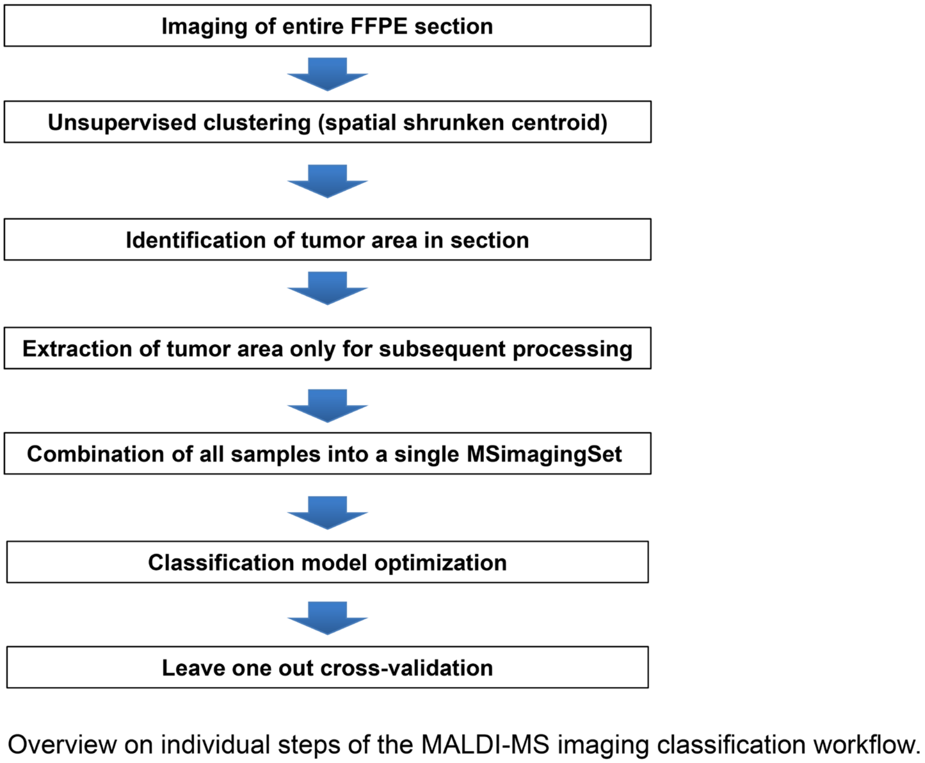

Supplementary Figure 4: Workflow for imaging data processing. Overview on individual steps of the MALDI-MS imaging classification workflow.

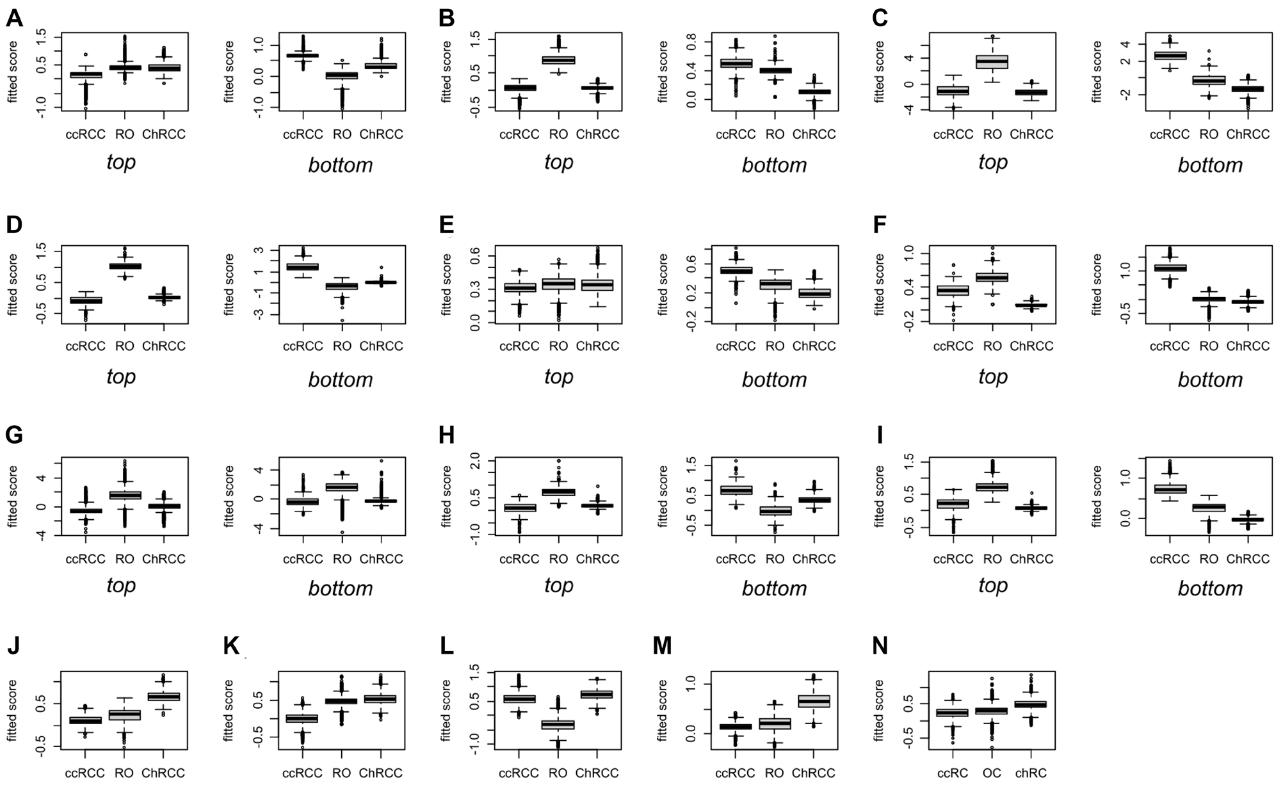

Supplementary Figure 5: Boxplot representation of scores from PLS-DA classification. Boxplot of PLS-DA fitted scores across all pixels in a patient sample. Scores for each individual testing condition (ccRCC, RO, ChRCC) for a given patient sample are plotted in one panel (**A**–**N**). Correlate with panels 3A–3N) in Figure 3.

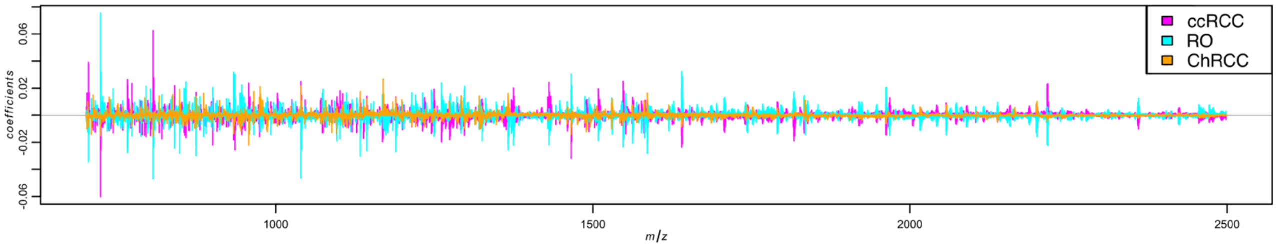

Supplementary Figure 6: PLS coefficients as a function of *m/z*. The diagram displays the impact of each detected *m/z* signal feature in imaging MS data from PLS-DA prediction (spectra are binned to 0.25 *m/z* bins). Positive coefficient indicates presence or higher abundance in the respective condition. Negative coefficient indicates absence or lower abundance of the *m/z* value in the respective condition (a list with the 100 most influential features can be found in Supplementary Material 1).

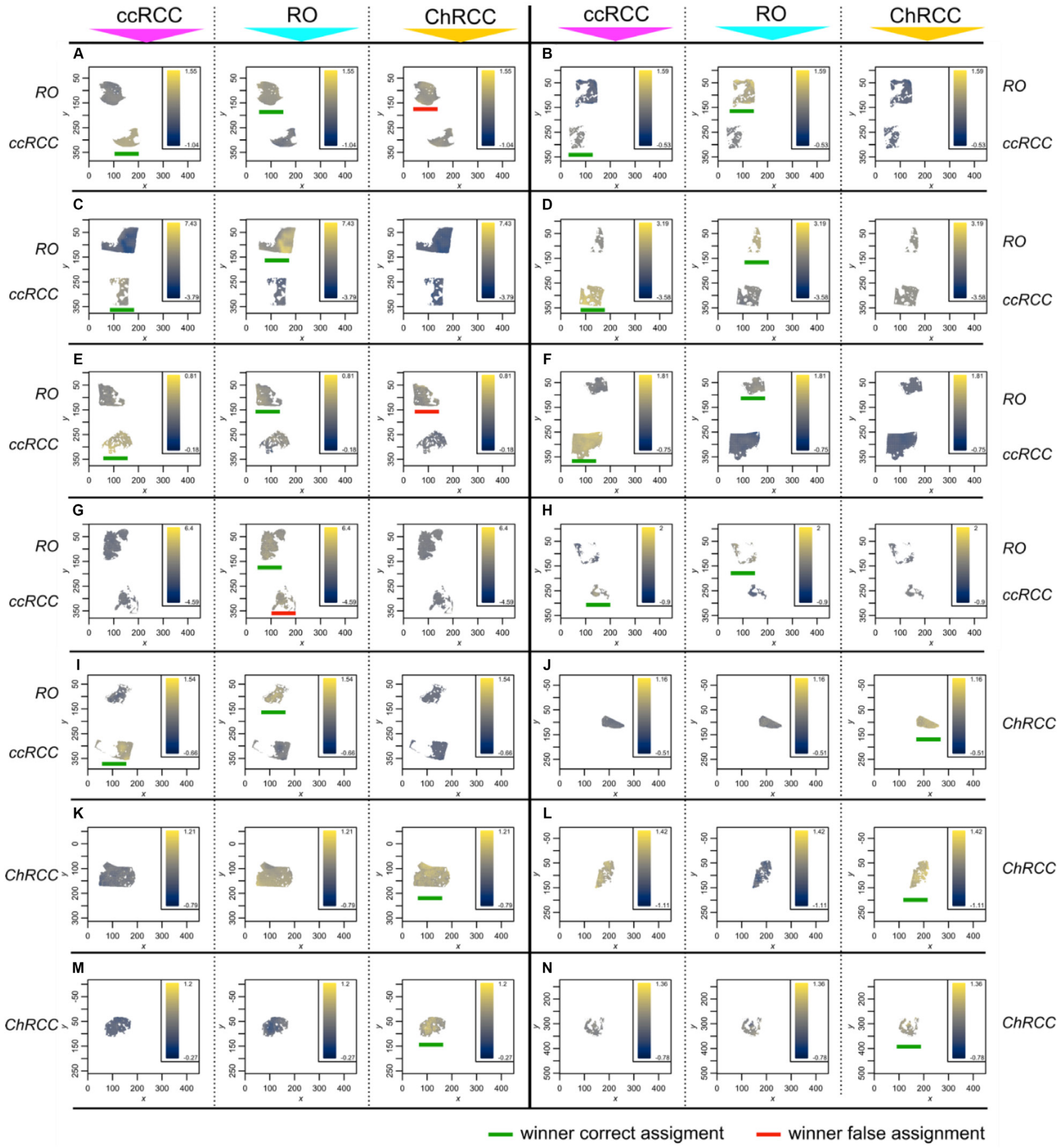

Supplementary Figure 7: Unprocessed version of Figure 3. Cross-validation results of PLS-DA classification.

Supplementary Table 1: Kidney tissue samples

FFPE

Cancer type	Clear cell renal carcinoma no.(ccRCC)	Grade	Gender	Leibovich score	Age	Renal Oncocytoma no. (RO)	Grade	Gender	Age	Chromophobe renal carcinoma no. (ChRCC)	Grade	Gender	Age
	427	4	M	8	69	270		M	73	835	3	M	63
	797	2	F	3	69	381		M	79	634	3	F	73
	370	3	M	5	59	119		F	51	756	3	F	26
	73	2	M	2	82	857		F	68	923	2	M	44
	999	2	M	5	50	527		M	82	264	4	M	39
	545	2	F	2	43	940		F	70				
	620	4	M	9	63	839		F	76				
	336	3	M	4	75	560		M	66				
	601	2	M	2	58	529		F	73				
	930	2	M	3	48	924		M	52				
	310	2	F	0	58	725		F	55				
	853	2	M	0	63								

Overview of FFPE sample number used in this study sorted according to the cancer subtype diagnosis (ccRCC, RO, ChRCC).

Supplementary Table 2: MSI sample overview

IMS Figure 3	Top	Bottom
A	839	427
B	119	370
C	527	620
D	270	73
E	529	545
F	560	999
G	940	797
H	857	601
I	381	336
J	634	
K	835	
L	264	
M	756	
N	923	

Sample overview on patient samples used for MALDI MSI analysis.

Supplementary Table 3: MSI median of fitted scoring

		Pixel median values					Median absolut deviation			
**Pannel**	**Position**	**ccRCC**	**RO**	**ChRCC**	**Winner**	**Pathologist diagnosis**	**ccRCC**	**RO**	**ChRCC**	**Diff. to second highest score**
A	**top**	0.1711	0.4159	0.4113	RO	RO	0.1204	0.0762	0.1474	0.0046
	bottom	0.6520	0.0364	0.3110	ccRCC	ccRCC	0.0627	0.1480	0.1016	0.6156
B	top	0.0750	0.8608	0.0657	RO	RO	0.1063	0.1435	0.0560	0.7950
	bottom	0.4987	0.4032	0.0992	ccRCC	ccRCC	0.0789	0.0506	0.0433	0.0954
C	top	−1.0940	3.4354	−1.3545	RO	RO	0.9283	1.4705	0.4987	4.7899
	bottom	2.5791	−0.3164	−1.3061	ccRCC	ccRCC	0.6037	0.6622	0.4149	2.8955
D	top	−0.0622	1.0247	0.0378	RO	RO	0.1223	0.1154	0.0403	0.9870
	bottom	1.3893	−0.3764	−0.0054	ccRCC	ccRCC	0.3803	0.3601	0.0509	1.7658
E	**top**	0.3133	0.3496	0.3363	RO	RO	0.0562	0.0659	0.0716	0.0133
	bottom	0.5013	0.3224	0.1841	ccRCC	ccRCC	0.0588	0.0890	0.0753	0.1789
F	top	0.3433	0.5687	0.0865	RO	RO	0.1068	0.1094	0.0231	0.4822
	bottom	1.0971	0.0065	−0.0906	ccRCC	ccRCC	0.1471	0.0981	0.0726	1.0906
G	top	−0.6215	1.5242	0.0451	RO	RO	0.4375	0.7039	0.3552	1.4791
	bottom	−0.3673	1.6751	−0.3266	RO	ccRCC	0.5359	0.6621	0.2159	2.0423
H	top	0.0786	0.7187	0.1868	RO	RO	0.1870	0.1716	0.0616	0.5318
	bottom	0.6651	−0.0372	0.3495	ccRCC	ccRCC	0.1936	0.1854	0.1178	0.7023
I	top	0.2093	0.7147	0.0811	RO	RO	0.1692	0.1671	0.0388	0.6336
	bottom	0.7213	0.2965	−0.0148	ccRCC	ccRCC	0.1304	0.1096	0.0441	0.4248
J		0.1030	0.2432	0.6535	ChrCC	ChRCC	0.1058	0.1570	0.1257	0.4104
K		−0.0056	0.4705	0.5442	ChrCC	ChRCC	0.1396	0.1038	0.1512	0.0737
L		0.5634	−0.3247	0.7352	ChrCC	ChRCC	0.1649	0.2135	0.1890	0.1718
M		0.1427	0.2096	0.6545	ChrCC	ChRCC	0.0709	0.1466	0.1636	0.4450
N		0.2312	0.3089	0.4746	ChrCC	ChRCC	0.1461	0.1328	0.1374	0.1656

Bold represents: difference < 0.05. Bold Italic represents: misassignment. Median values of classification fitted scores. Panel column refers to panels in Figure 3. Top and bottom indicate sample positions within the panels of Figure 3.
